# A unique cell population expressing the Epithelial-Mesenchymal Transition-transcription factor Snail moderates microglial and astrocyte injury responses

**DOI:** 10.1093/pnasnexus/pgad334

**Published:** 2023-10-12

**Authors:** Cheryl Clarkson-Paredes, Molly T Karl, Anastas Popratiloff, Robert H Miller

**Affiliations:** Department of Anatomy and Cell Biology, School of Medicine and Health Sciences, George Washington University, 2300 Eye Street NW, Ross 735, Washington, DC 20052, USA; Nanofabrication and Imaging Center, The George Washington University, 800 22nd Street NW, Washington, DC 20052, USA; Department of Anatomy and Cell Biology, School of Medicine and Health Sciences, George Washington University, 2300 Eye Street NW, Ross 735, Washington, DC 20052, USA; Department of Anatomy and Cell Biology, School of Medicine and Health Sciences, George Washington University, 2300 Eye Street NW, Ross 735, Washington, DC 20052, USA; Nanofabrication and Imaging Center, The George Washington University, 800 22nd Street NW, Washington, DC 20052, USA; Department of Anatomy and Cell Biology, School of Medicine and Health Sciences, George Washington University, 2300 Eye Street NW, Ross 735, Washington, DC 20052, USA

**Keywords:** reactive astrocytes, reactive microglia, neuroinflammation, Snail

## Abstract

Insults to the central nervous system (CNS) elicit common glial responses including microglial activation evidenced by functional, morphological, and phenotypic changes, as well as astrocyte reactions including hypertrophy, altered process orientation, and changes in gene expression and function. However, the cellular and molecular mechanisms that initiate and modulate such glial response are less well-defined. Here we show that an adult cortical lesion generates a population of ultrastructurally unique microglial-like cells that express Epithelial-Mesenchymal Transcription factors including Snail. Knockdown of Snail with antisense oligonucleotides results in a postinjury increase in activated microglial cells, elevation in astrocyte reactivity with increased expression of C3 and phagocytosis, disruption of astrocyte junctions and neurovascular structure, increases in neuronal cell death, and reduction in cortical synapses. These changes were associated with alterations in pro-inflammatory cytokine expression. By contrast, overexpression of Snail through microglia-targeted an adeno-associated virus (AAV) improved many of the injury characteristics. Together, our results suggest that the coordination of glial responses to CNS injury is partly mediated by epithelial-mesenchymal transition-factors (EMT-Fsl).

**Significance Statement**

Injuries to the adult nervous system trigger a cascade of responses in glial cells that either accentuate initial damage or facilitate recovery. How those responses are regulated is not well understood. Here, we show that a cortical injury induces a localized population of cells that express the transcription factor Snail that has been implicated in repair in other tissues. Reducing the expression of Snail increases glial reactivity and accentuates secondary neuronal damage. By contrast, overexpression of Snail reduces glial reactivity and mitigates secondary neuronal damage. Based on these observations, we propose that Snail is part of a regulatory pathway that limits the spread of injury-induced damage in the adult CNS.

## Introduction

Glial responses to CNS insults and neuropathologies share several common characteristics (1, 2). After an injury, microglia and astrocytes become reactive and undergo modulation of multiple physiological functions to acquire molecular, phenotypic/morphological, and metabolic profiles that may be either beneficial or detrimental to recovery (3, 4). Following an insult, microglia rapidly become activated and undergoes changes in gene expression profiles (5), metabolic states (6), and cytokine production and release (7). Microglia also appears to influence subsequent astrocytic responses in a neuroinflammatory environment, inducing a powerful neurotoxic astrocytic phenotype through the secretion of cytokines including Il-1a, TNF (tumor necrosis factor), and C1q (8). These neurotoxic astrocytes express many altered functions affecting phagocytic capacity, synapse formation, and function, and promoting neuronal death (8).

A common thread that links cell phenotypic transformation after injury in other systems is the epithelial-mesenchymal transition (EMT). The EMT program is a set of multiple and dynamic transitional states between the epithelial and mesenchymal cell phenotypes, reflecting the delicate balance of transcriptional drivers and suppressors of EMT (9). This plasticity of cellular transformation has been considered a crucial driver in adult in wound healing (10, 11), fibrosis (12, 13), and cancer (14, 15). In the CNS, EMT expression is largely restricted to early development (9, 16) and glioblastomas (17, 18); while in the peripheral nervous system, the reprogramming of Schwann cells to an invasive EMT-dependent mesenchymal phenotype that participates in recovery from injury has been well described (19). The expression of EMT in response to injury or tumorigenesis is closely linked to inflammation in other tissues where it stimulates the production of inflammatory factors that in turn act as a potent inducer of EMT (20). For example, in the mesentery, EMT mediates the transdifferentiation of mesothelial cells into macrophages and subsequent changes in their cytokine production (21, 22).

The induction of EMT is triggered in response to environmental signals (23) including inflammation and engages transcriptional regulators, and downstream effectors such as transforming growth factor beta/TGF-β (24–27), Wnt/β-catenin (28–30), Notch (31–33), miRNAs (34–37), and growth-factor receptor signaling (38, 39). These factors activate signaling pathways leading to either expression or modification of the main families of EMT-transcription factors such as SNAI (SNAI1/Snail and SNAI2/Slug), zinc finger E-box binding homeobox/ZEB (ZEB1 and ZEB2), and TWIST (TWIST1 and TWIST2). While Snail is highly expressed during embryonic development (40, 41), reactivity in adult tissue is sparse (42). The transcription factor Snail also participates in EMT-independent functions including protecting cells from death induced by the loss of survival factor or by apoptotic stimuli (41). It has also been proposed that cytokine-induced expression of Snail reduces pro-inflammatory responses in macrophages (43). Here we assess the re-expression of the main EMT-transcription factors after injury to the adult CNS and identify a unique cell population expressing Snail. The Snail+ cells share characteristics of microglia/macrophage and reducing expression of Snail with antisense oligonucleotides significantly increases pro-inflammatory responses including elevation of astrocyte phagocytosis and disruption of astrocyte coupling. By contrast, overexpression of Snail from microglia/macrophage promoters decreases pro-inflammatory cytokines secretion, switching reactive neurotoxic astrocytes to a more neuroprotective role. Furthermore, injury-induced neuronal apoptosis was significantly increased, and the number and size of synapses significantly decreased following Snail after Snail silencing (ASO) treatment, while Snail overexpression promoted the preservation of cortical synapses in the injured area and increased the terminal pre- and postsynaptic glial enwrapping. These findings suggest that regulation of microglia Snail expression in the adult CNS influences the astrocytic responses after injury. Manipulation of these interactions would provide beneficial effects for CNS repair and opportunities for developing new therapeutic strategies for neuroinflammatory/neurodegenerative diseases.

## Results

### EMT-activating transcription factors are expressed in the adult CNS in response to injury

**Fig. 1.**
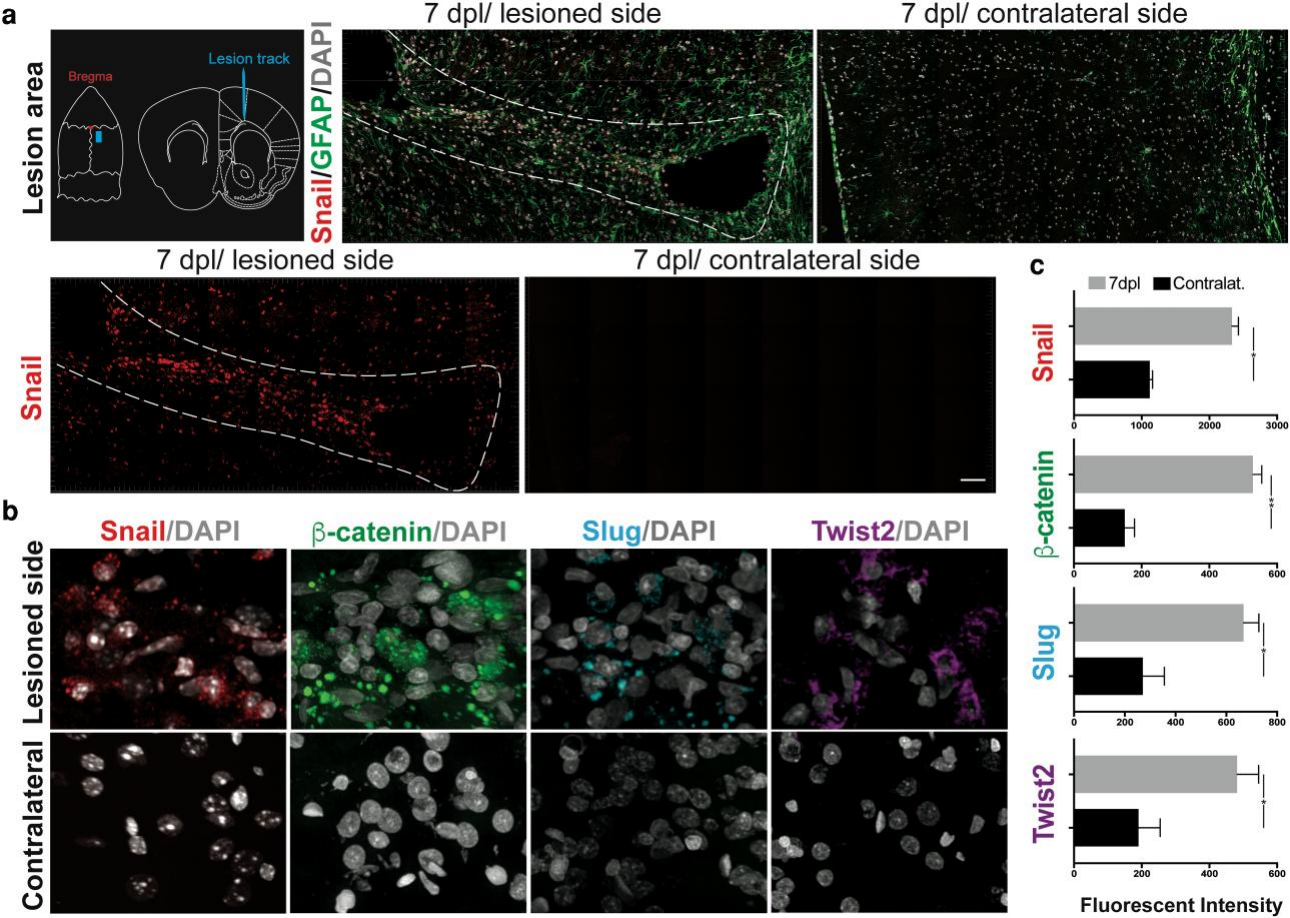
Expression of EMT-effectors and EMT-inducing transcription factors after injury in the adult cortex. a) Representative diagram showing the lesion cortical region, and immunofluorescence tile images of lesioned adult rodent cortex, showing expression of Snail EMT-associated transcription factor 7 days after a cortical injury (7 dpl), and the contralateral side (control). b) High-magnification images showing expression details for EMT-associated transcription factors and signaling pathway markers at 7 dpl. Immunolabeling studies were run simultaneously, using the same imaging acquisition parameters in all cases. c) Quantification of fluorescent intensity using HyD detectors and single-photon counting showed a significant increase in the expression ratio for all EMT-associated transcription factors compared to the contralateral side (Wilcoxon matched-pairs, *P*-value * ≤ 0.05; ** ≤ 0.01). Scale bars: 50μm (a); 10 μm (b).

EMT-transcription factors (EMT-TFs) play essential roles during embryonic CNS development (44), while their expression in the adult CNS has been described largely in relation to oncogenic processes. Based on their involvement in inflammatory and wound-healing responses in other tissues (9, 21), we assessed the expression of EMT-TFs in an adult cortical stab wound model of CNS injury. At seven days postinjury immunohistochemical analysis revealed that the primary EMT-TFs including Snail, Slug, and Twist as well as the EMT effector β-catenin were expressed along the lesioned zone, including labeling in most cortical layers (Fig. 1a, b). All EMT-TFs examined showed a higher level of expression in the lesion core compared to peri-lesioned areas, while in the control cortex (contralateral side), no significant expression of EMT-TFs or β-catenin was detected. Surprisingly, neither EMT-TFs nor β-catenin expression was detected in the proliferative layers near the cerebral ventricle or other adjacent areas, suggesting the induction of a local expression of the EMT-TFs postinjury. The cellular localization of EMT-TFs at 7 dpl was predominantly cytoplasmic rather than nuclear while β-catenin showed a mixed nuclear/cytoplasmic localization (Fig. 1b). Quantitative fluorescent signal analyses using segmentation and surface creation of the high-resolution confocal images suggested that β-catenin and the transcription factor Snail had high expression in cells in the lesioned regions (Fig. 1c) suggesting they have a greater injury-induced response in the adult CNS.

### Snail+ cells share molecular and ultrastructural characteristics with a subset of microglia

**Fig. 2.**
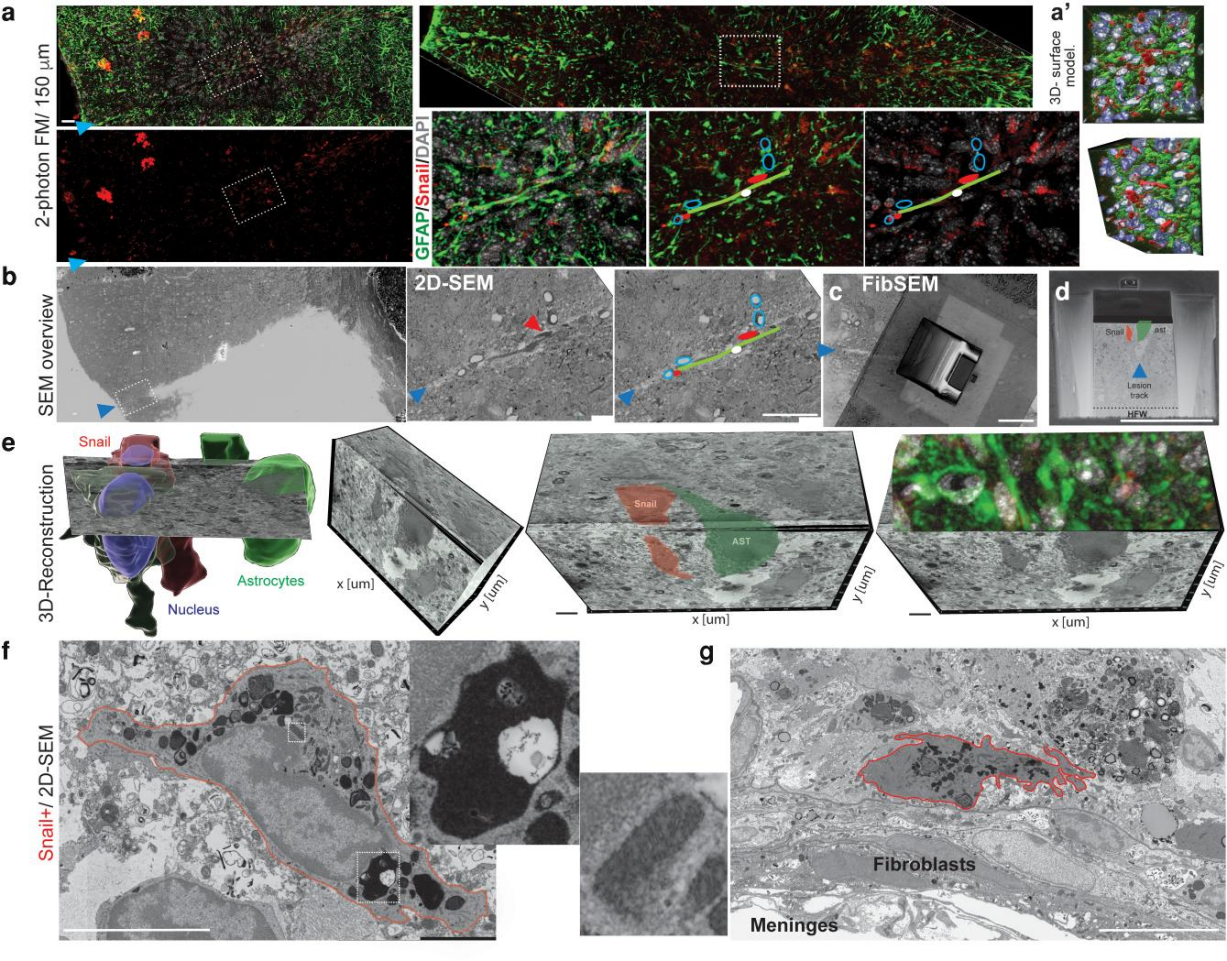
Characterization of Snail+ cells that develop after adult cortical injury. The identity of Snail-expressing cells was assessed at 7 dpl using a combination of correlated fluorescence microscopy (a-a′) and 2D (b)/3D FibSEM approaches (c–e) in the same field. a) Two-photon confocal images of a thick section (150 μm/max. projection) showing the localization of the lesion (arrowheads) and visualization of GFAP expression to identify reactive astrocytes, Snail expression, and nuclei staining. Diagram showing the selected FibSEM location, where blood vessels (circles), astrocytes processes, and lesion track established biological fiducial points for correlation with SEM. a′) surface model 3D-fluorescent volumetric reconstruction identifying the position of the Snail+ cell nuclei in the z-plane. b) Low-magnification 2D-SEM tile images showing the partial resin-exposition of the sample in (a) following EM processing. Subsequent identification of the location in the SEM that was confirmed by fluorescent data, FibSEM trenches, and block-face SEM imaging (c–d), showing the z-location of the target Snail+ cell (d), were performed (203 sections, z: 40 nm). e) Multiple merged angles-view showing the fluorescent and 3D-SEM correlation identifying a Snail+ cell. f) Example of Snail+ cell in 2D-SEM displaying cytoplasmic and nuclear ultrastructural characteristics of CNS phagocytic cells. g) 2D-SEM images showing differences between Snail+ cells and meningeal fibroblasts in the lesion area. Scale bars: low mag light microscopy (LM)/SEM: 30 μm. (f) 5 μm. (g) 10 μm.

Multiple cell types can express Snail, which plays a role in EMT-dependent and -independent functions including immune regulation, cell stemness, movement, and survival (41, 45, 46). To identify the characteristics and ultrastructural signature of the injury-induced CNS Snail+ cells, we developed a correlative fluorescent light and high-resolution volumetric electron microscopy workflow (Fig. 2). At 7 dpl, Snail+ cells were localized in the injury track interspersed with glial fibrillary acidic protein (GFAP)+ reactive astrocytes but were not themselves GFAP+ (Fig. 2a). To determine whether Snail+ cells exhibited a singular ultrastructural profile, we correlated the labeled two-photon image from thick sections (Fig. 2a-a′) to the tile-imaging SEM 2D-data from the same precise area (Fig. 2b). Following fiducial points including the lesion track, blood vessels, and nuclei distribution (Fig. 2b), we aligned the 2D images from different modalities to identify/select a Snail+ cell (region of interest/ROI), deposited a protective layer over the ROI (surface Snail cell), milled the trench (Fig. 2c), and performed the focus ion beam 3D-SEM serial sectioning (Fig. 2c, d). Volumetric SEM reconstruction of Snail+ cells revealed a novel ultrastructural profile, with dark cytoplasm previously identified as a subtype of microglia (dark microglia (47)) but with elongated, heterochromatic, and less invaginated, smoothly contoured nuclei (Fig. 2e, f). The cytoplasm contained little endoplasmic reticulum and a high number of mitochondria that were highly variable in size, present in patches, with a dark appearance (inset Fig. 2f). The most apparent characteristic of Snail+ cells was the high density of large inclusions filling the whole cytoplasm with dense core structures resembling lysosomal inclusions bodies, the likely product of phagocytosis (Inset Fig. 2f, Supplementary Fig. S1), consistent with the hypothesis that Snail+ cells are a subtype of microglia/macrophages recruited after CNS injury.

**Fig. 3.**
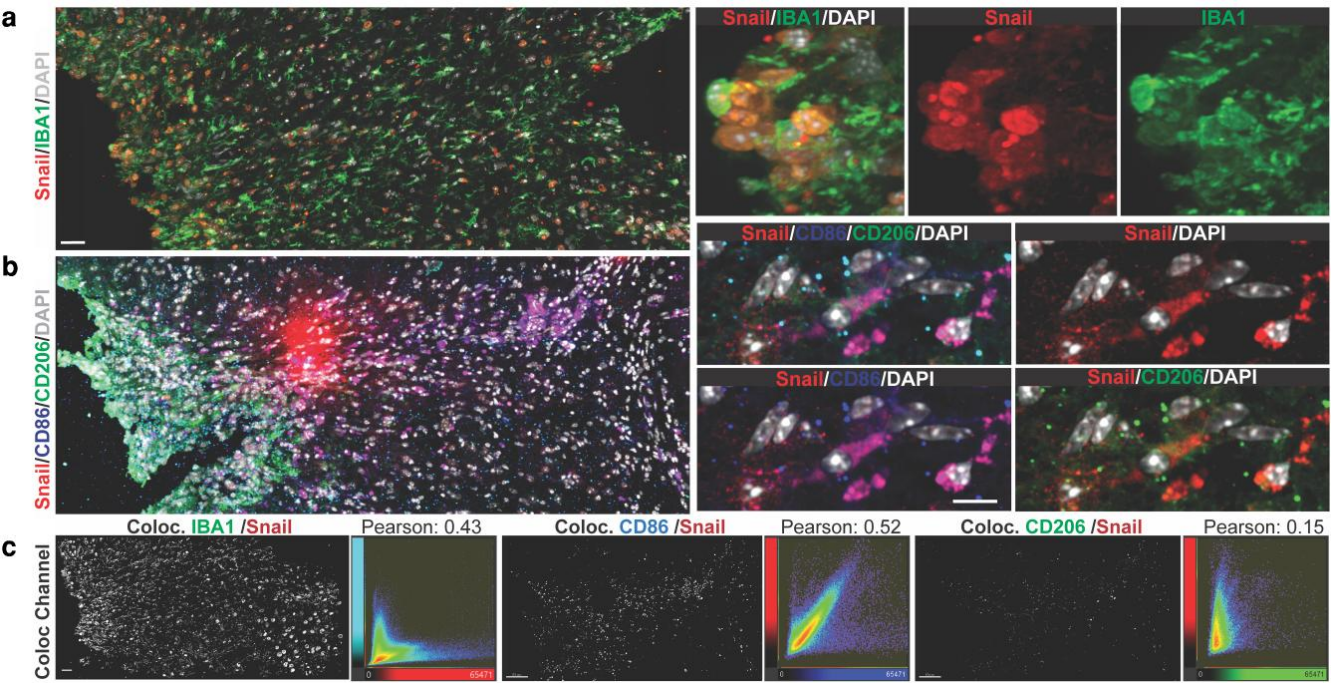
Snail is expressed in microglial/macrophage cells after CNS injury. Insults to the adult CNS affect multiple microglia/macrophage responses and the homeostatic perturbations alter their phenotype including the expression of transcription factors. a) Representative confocal fluorescent tile images of cortical lesions after 7 days postinjury labeling EMT-TF Snail, IBA1, and DAPI. High magnification showing a subset of Snail/IBA1 positive cells in the lesioned area. b) Confocal tile-imaging for Snail, M1 marker (CD86), M2 marker (CD206), and DAPI in the lesioned cortex. High magnification shows the strong co-localization of Snail with CD86 and a weaker colocalization of Snail and CD206 postinjury. c) Colocalization multichannel analysis and 2D-histogram plots exhibited a significant overlapping of Snail/IBA1 channels and Snail with M1 marker labeling, while M2 marker and Snail labeling showed a non-significant colocalization coefficient. Scale bars: low-mag: 30 μm, high-mag: 10 μm.

Phenotypic analyses with a range of cell-type-specific markers indicated no colocalization of Snail with markers for astrocytes, pericytes, endothelial cells, or neurons (data not shown). In addition, ultrastructural analyses demonstrated profound morphological differences between Snail+ cells and meningeal fibroblasts present in the lesioned area (Fig. 2g). In addition, Cha et al. (48) demonstrated that after photothrombosis AKAP12 expression in fibroblasts inhibits SNAI1, making it unlikely that Snail+ cells are a phenotypic transition of meningeal fibroblast (48). By contrast, the Snail+ cells shared characteristics with microglia. Activated microglial/macrophage cells upregulate IBA1 expression and exhibit a range of polarization states (49). We found a strong colocalization between Snail and the IBA1 marker after 7 dpl (Fig. 3a, c). Using quantitative intensity fluorescence data and Pearson's correlation coefficient measurement, we found that Snail colocalized with both CD86 and CD206 (Fig. 3b, c), indicators of pro and anti-inflammatory functions, respectively, although the coefficient of correlation was stronger with CD86, suggesting these cells were the primary source of Snail postinjury. CD86 is also considered a CNS marker for the M2b macrophage subtype (50, 51), the subtype that secretes alternatively pro-inflammatory and anti-inflammatory cytokines, thus, performing some immunoregulatory functions (52). Microglia M2b also expresses CD86 (53), consistent with the concept that Snail+ cells are microglia/macrophages with an immune profile that are induced in the adult CNS postinjury.

### Modulation of Snail in vivo after cortical injury

**Fig. 4.**
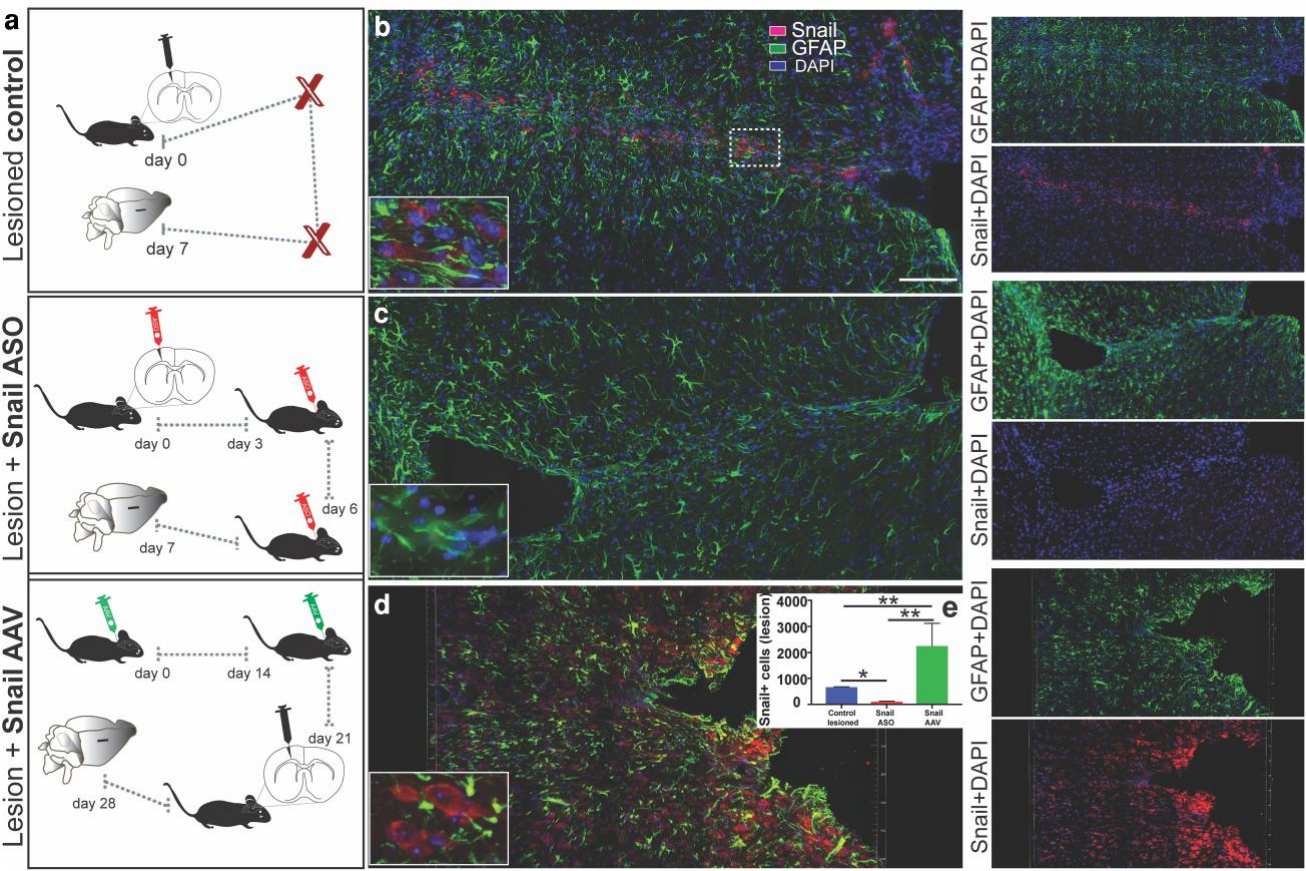
*In vivo* regulation of Snail expression in microglia/macrophage after injury in the adult CNS. a) Outline of surgical approach for lesioned, Snail-ASO, and Snail-AAV groups. All animals were perfused at 7 dpl. Animals were injected with Snail-AAV 28 days prior to the lesion to ensure appropriate expression. The Snail-AAV group ultimately matched the age and dpl of lesioned controls and Snail-ASO at sample collection. b) Confocal tile images showing expression of reactive astrocytes and Snail+ cells associated with the lesion track at 7 dpl. c) Tile images of the lesioned area showing the silencing of Snail expression using Snail-antisense oligonucleotide gapmers at 7 dpl. d) Representative confocal imaging section of a lesioned cortex after Snail-AAV-PHP.eB vector delivery in the cisterna magna, exhibiting overexpression of the Snail EMT-TF in the F4/80 microglia/macrophage after 7 dpl. e) Quantification of the number of positive Snail cells in the lesioned control, ASO, and Snail overexpression (AAV). Data presented as mean ± SEM, Kruskal–Wallis, multiple comparisons *P ≤ 0.05 **P ≤ 0.005. Scale bars: 100 μm.

To knock down or over-express Snail in the lesion area, in vivo local injections of Snail ASO or an adeno-associated virus (AAV) were used. Adult littermates were divided into four groups: nonlesioned, lesioned+ vehicle, lesioned+ Snail-ASO, and lesioned+ Snail-AAV (Fig. 4a). In the lesioned group, Snail was expressed in cells along the entire lesion track with greater expression in the center of the lesion as revealed by coronal plane analysis (Fig. 4b). Knockdown of Snail expression through ASO delivery (2 μL, 53.45 ng/μL) was highly effective with a dramatic decrease in Snail expression 7 days after Snail-ASO injection (Fig. 4c). In addition, quantification of Snail+ cells in the region of the lesion showed a significant decrease of 84% compared to injury controls (Fig. 4e) with the remaining positive cells expressing significantly lower fluorescent intensities of labeling (−50%). In controls, similar delivery of scrambled ASO had no effect on Snail expression indicating the specificity of Snail-ASO. To locally over-express Snail in the lesion area, an AAV approach was utilized. Delivery 28 days before the injury of an AAV-PHP.eB targeted to F4/80 promoter (mCherry tagged) resulted in an increased overexpression of Snail in vivo (Fig. 4d) after 7 days postinjury. Quantification of expression demonstrated an in vivo overexpression of Snail greater than 300% compared to the lesioned group (Fig. 4e).

### Modulation of Snail expression influences microglial response and cytokine expression

**Fig. 5.**
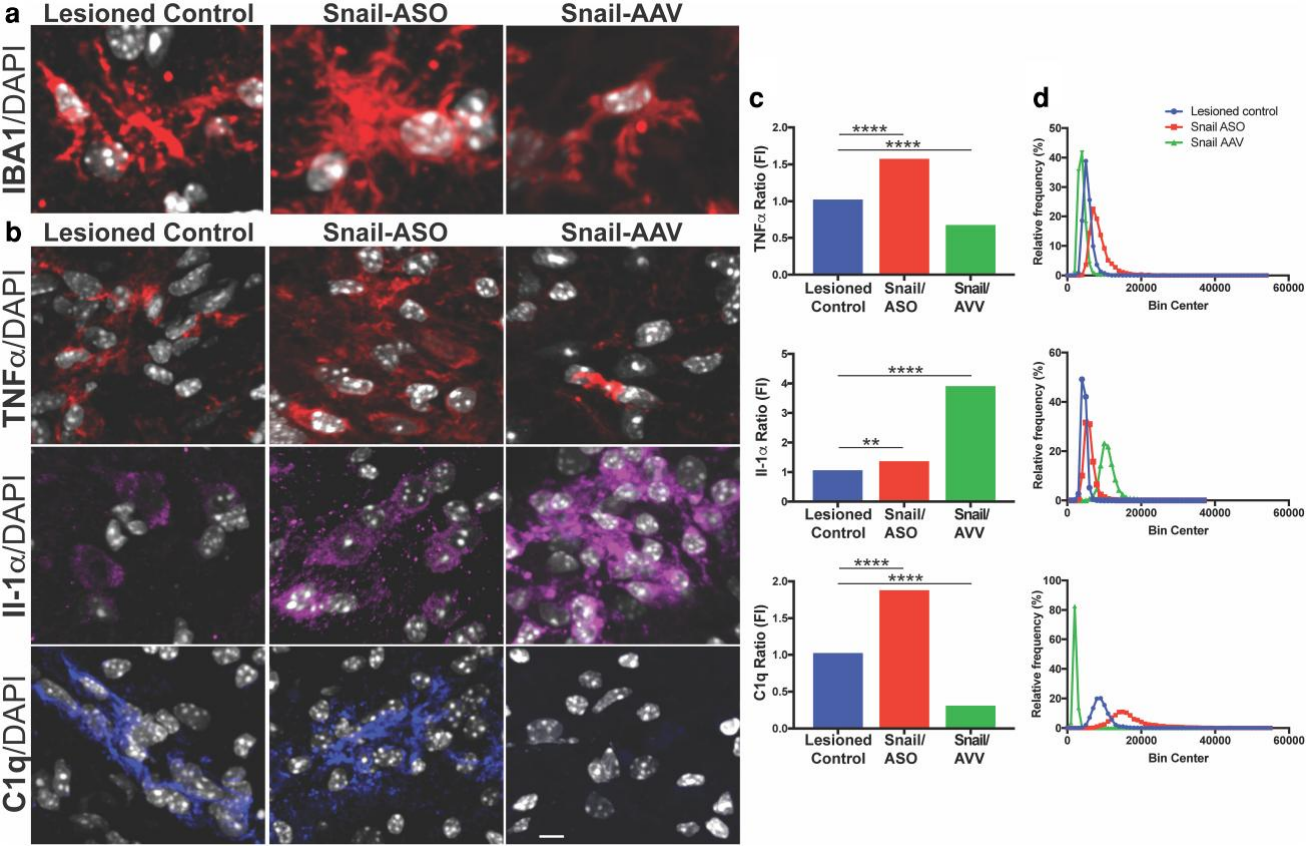
Snail expression regulated CNS insult-induced pro-inflammatory cytokines. Confocal images of lesioned (a), Snail-ASO, and Snail-AAV lesioned treated samples, labeled for TNFα, Il-1α, C1q, and DAPI at 7 dpl (b). Quantification was performed in the lesion and peri-lesioned area (up to 500 microns away from the needle track). Note the significant increase in all cytokines after Snail ASO and the TNFα and C1q decrease after Snail-AAV treatment (c) Plots showing the intensity fluorescent quantification for TNFα, Il-1α, and C1q; data presented as a ratio, Mann–Whitney two-tailed, **P < 0.005 ****P < 0.0001. d) Relative frequency histograms showing shifts in the intensity distribution per group after Snail-ASO and Snail-AAV treatment. Scale bars: high mag: 7 μm.

In other conditions, the expression of Snail results in immune modulation (54, 55). To determine whether following injury to the adult CNS Snail influenced microglial/macrophage activation, we compared the responses of IBA1+ cells in the lesioned group, following ASO knockdown and after AAV overexpression of Snail. In lesioned group, an accumulation of reactive microglia/macrophage and changes in cellular morphology from resting to more ameboid phenotype was seen (Fig. 5a). Following Snail knockdown with ASO treatment, IBA1 positive cells exhibited a more pronounced reactive phenotype with larger cell bodies and increased cellular complexity (Fig. 5a). By contrast following overexpression of Snail with AAV delivery, IBA1 cells showed small cell bodies and a delicate ramified morphology, with thin branches consistent with a surveilling state. These findings suggest that Snail expression may modulate microglia activation by regulating the phenotypic switch between surveilling and activated states.

To determine if modulation of Snail expression influenced the expression of pro-inflammatory cytokines that trigger an A1-astrocyte phenotype (8), levels of TNFα, Il-1α, and C1q were compared between control, lesioned-control, lesioned+ Snail-ASO, and lesioned+ Snail-AAV samples. Compared to controls, the lesioned group had elevated expression of TNFα, Il-1α, and more predominantly C1q in the lesion and peri-lesioned areas at 7 dpl. Treatment with Snail ASO dramatically increased the expression of these cytokines (Fig. 5b). Quantification by fluorescent intensity revealed an increase in TNFα expression of 57%, while IL-1α was increased by 28% and C1q was increased by 86% compared to the lesioned group (Fig. 5c). The number of cells expressing TNF-α and IL-1α also increased after Snail ASO treatment (Fig. 5d). C1q, the most abundant cytokine at 7 dpl, showed the highest upregulation after Snail silencing (Fig. 5c, d). By contrast, overexpression of Snail by AAV delivery resulted in a 35% decrease in TNF-α and a 71% decrease in C1q compared with the lesioned group (Fig. 5b–d). Conversely, Il-1α increased after Snail overexpression by 385% but with fewer positive cells (21%) compared to lesioned animals. These results indicate that Snail expression alters the cytokine profile that accompanies CNS injury.

### Changes in Snail expression influence astrocyte phenotypes, phagocytic activity, and glycogen accumulation after injury

**Fig. 6.**
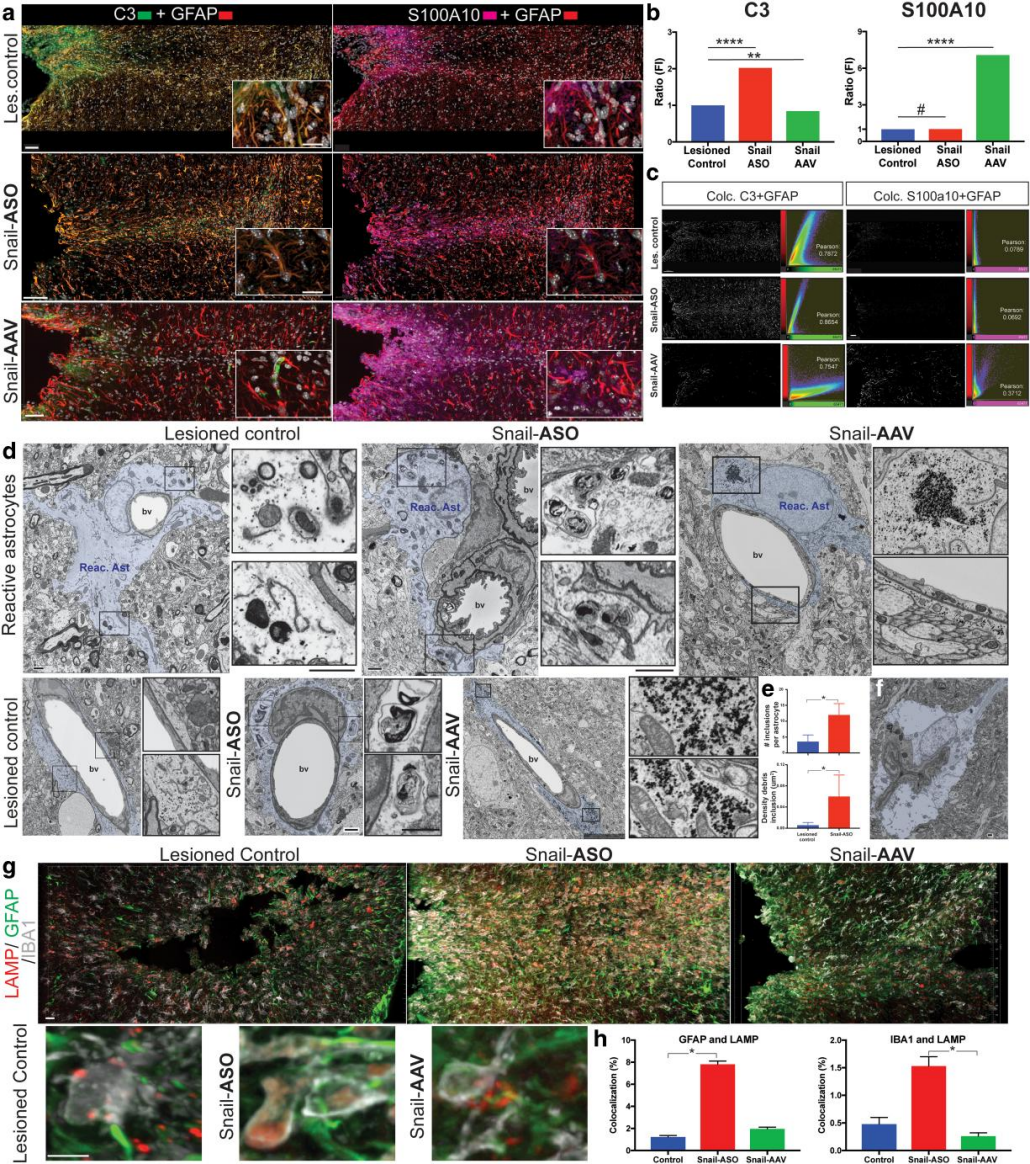
Snail modulation altered the molecular profile of reactive astrocytes, enhancing phagocytic activity. Confocal tile images from the lesioned area (up to 500 μm from the injury track) showing the expression of markers associated with neurotoxic astrocytes (C3+), neuroprotective astrocytes (S100A10+), and GFAP+ reactive astrocytes in the injured area at 7 dpl, following Snail-ASO, and Snail-AAV overexpression. Note the increase in C3/GFAP marker expression after Snail-ASO and the strong decrease after Snail-AAV treatment. No significant change in S100A10+ labeling was detected following Snail-ASO, but a significant increase was seen after Snail-AAV treatment. b) Mean fluorescent intensity quantification for C3 and S100A10 showed a significant increase (102%) in the expression of C3 after Snail-ASO. Snail-AAV decreased C3 expression and a dramatic increase in S100A10 labeling in the lesioned region. Data presented as intensity mean ± SD, C3: Kruskal Wallis posthoc comparisons **P < 0.005; ****P < 0.0001; S100A10: Kruskal Wallis + posthoc comparisons ****P < 0.0001. c) A quantitative measure of colocalization multichannel analysis based on the pixel-intensity correlation between GFAP labeling and C3 showed a strong correlation in all analyzed groups, while the correlation for GFAP and S100A10 marker was minimal for lesioned and Snail-ASO groups and increased colocalization after Snail-AAV treatment. d) Representative high-resolution 2D-SEM images of activated (lesioned control), phagocytic (Snail-ASO), and highly glycogen-content (Snail-AAV) astrocytes surrounding blood vessels in lesioned areas. Astrocytes from all injury groups showed extended and thicker end-feet processes associated with blood vessels than noninjured controls. Snail-ASO treatment increased the frequency of the phagocytic astrocytes, with significant increases in the size of phagocytic content, while Snail-AAV treated astrocytes had an increase in the glycogen content filling in cytoplasm, main, and fine distal processes. e) Quantitative analysis indicates that phagocytic inclusions were more prominent after Snail ASO treatment, with increases in the number and density compared to the lesioned group. Data are presented as the mean ± SD, Mann–Whitney, two-tailed *P* = 0.0159. f) Representative example of reactive astrocyte in the Snail-ASO group showing aberrant behavior including collapsing a blood vessel lumen. EM Scale bars, (a) low-mag: 50 μm, high-mag: 20 μm. g) Representative tile confocal images and high-magnification insets of lesioned, Snail-ASO, and Snail-AAV groups analyzing alterations in autophagy marker expression/location (LAMP) inside of reactive astrocytes (GFAP) and microglia/macrophage (IBA1). h) Plot graphs showing the percentage of colocalization for LAMP/GFAP and LAMP/IBA1 in all analyzed groups (Kruskal Wallis, multiple comparisons *P* value* ≤ 0.05). LM scale bar: low mag: 20 μm; high- mag: 8 μm.

The cytokines whose expressions are altered by modulating Snail levels have been implicated in affecting astrocyte phenotypes (8). To determine whether changes in Snail expression induced by ASO or AAV treatment altered the phenotype of reactive astrocytes after injury, we compared their expression of phenotypic markers and phagocytic activity in the lesioned group and after Snail ASO or changes in glycogen accumulation after Snail-AAV treatment (Fig. 6). In lesioned tissue (7 dpl), reactive astrocytes identified by high levels of GFAP labeling and hypertrophy, expressed moderate levels of the pro-inflammatory marker (C3+) and a low level of S100A10+ (Fig. 6a–c). By contrast, following Snail ASO treatment, the level of astrocyte activation was significantly higher (Fig. 6a), with an increased density in reactive astrocytes in the lesion area, a significant increase (+102%) in the expression of C3+/GFAP+ astrocytes (Fig. 6b, c) and no significant change in the expression of S100A10+ (Fig. 6b, c). No differences in marker expression were detected in samples treated with scrambled ASO suggesting that neurotoxic astrocyte reactivity is enhanced in the absence of Snail. Consistent with this hypothesis, overexpression of Snail through AAV delivery resulted in a reduction in the expression of C3 in the lesioned area (13%) compared to the lesioned group and a dramatic increase (708%) in the expression of S100A10. To quantify the cellular identity of C3 expression, a colocalization signal analysis was performed and showed a strong Pearson's correlation coefficient for all groups between GFAP and C3, while a weaker correlation was detected for S100A10 for lesioned and Snail-ASO groups (Fig. 6c) with higher colocalization after Snail-AAV treatment. These results suggest that Snail expression in microglia/macrophages influences the anti-inflammatory glial responses after injury in the adult CNS.

One astrocytic characteristic that is modulated by microglia and correlated with a reactive neurotoxic phenotype is the level of phagocytosis (8). To determine whether changes in Snail expression altered astrocyte phagocytosis after injury the phagocytic responses were compared between lesion controls and after ASO and AAV treatment using high-resolution and large-area 2D-SEM approaches. In the lesioned group, reactive astrocytes were evident (Fig. 6d, e), with hypertrophic processes extending around the blood vessels and neuropil of the lesioned area. By contrast, after the ASO knockdown of Snail, the number of reactive astrocytes, complexity, and size of inclusions/astrocytes were increased approximately 3fold, and their density was significantly higher (Fig. 6e). In extreme cases, astrocytes appeared to be attempting to engulf entire blood vessels with collapsed lumens (Fig. 6f) consistent with increased and dysfunctional phagocytic activity. Conversely, after AAV-induced overexpression of Snail, astrocytes exhibited a higher density of very small glycogen granules (puncta size) within the cytoplasm and fine distal processes interacting with pre and postsynaptic components in the neuropil, but no large storage bodies were found suggesting an increase of astrocyte glycogen mobilization. An increase in astrocytic glycogen mobilization is linked to a neuroprotective effect after injury (56, 57) while playing a vital role in supporting physiological brain functions (58). Consistent with changes in astrocyte phagocytosis, analysis of the expression of lysosomal associated membrane protein 1 (LAMP1), a lysosomal protein linked to cytotoxic activity (59) and responsible for the macromolecular degradation following phagocytosis and autophagy (60) was performed. 3D-Immunofluorescence analysis showed LAMP1 expression colocalizing with microglia and astrocyte markers in lesioned controls (Fig. 6g, h). Following ASO treatment, LAMP expression increased by 534% in GFAP+ astrocytes and 218% in the IBA1+ microglia/macrophage in the lesioned area, while following AAV treatment LAMP increased in astrocytes by 59% and decreased by 54% in IBA1+ cells (Fig. 6g, h). Together these data suggest that in the adult injured CNS, Snail expression in microglial-like cells modulates phenotypic and functional astrocyte characteristics.

### Expression of astrocyte connexins was altered by Snail

**Fig. 7.**
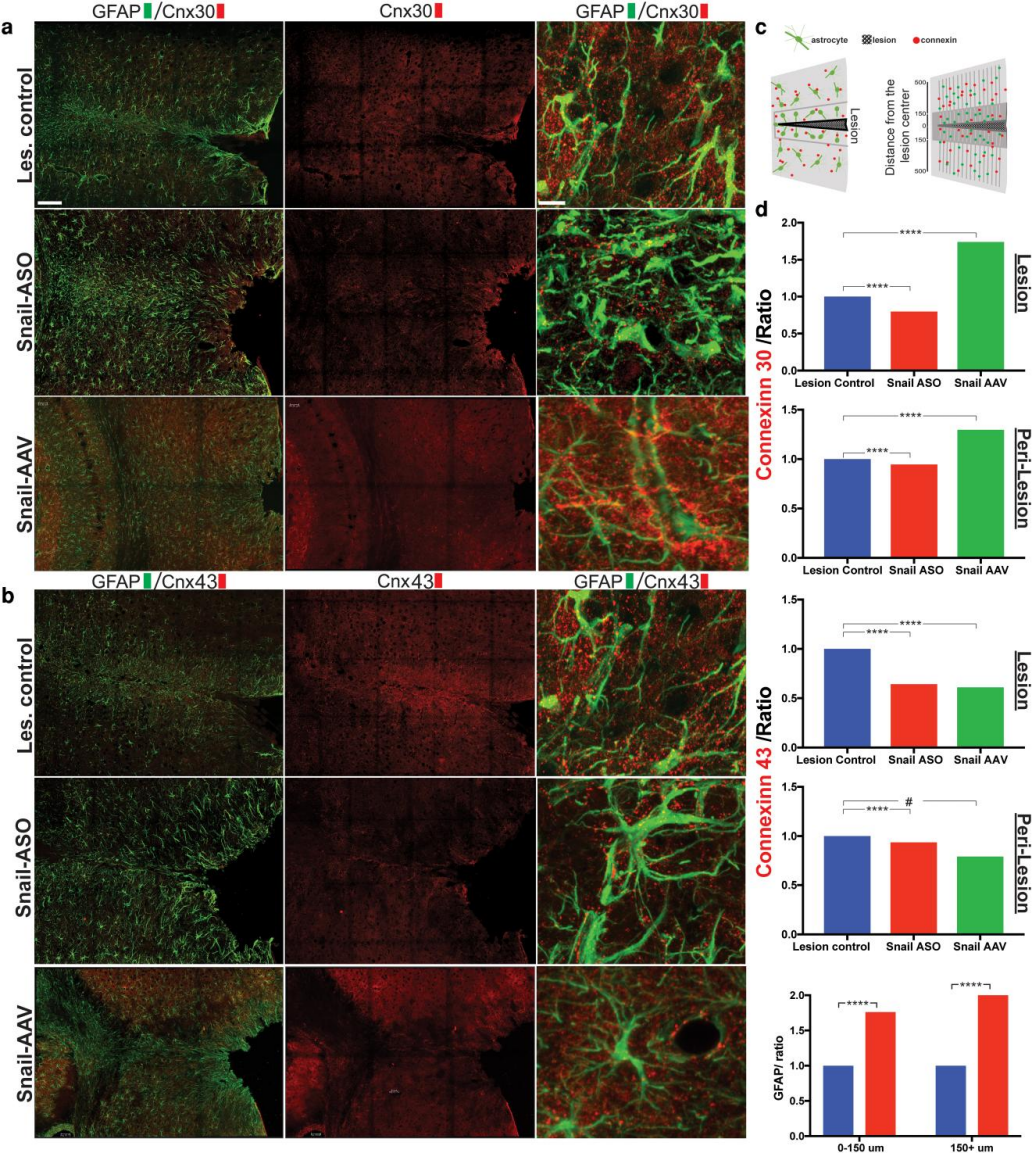
Astrocytic connexins expression was reduced in lesion and peri-lesion areas following Snail ASO. Representative confocal/two-photon tiled imaging of lesioned mouse neocortex stained for GFAP and a) astrocytic connexin 30 and b) Connexin 43, in the lesioned group, after Snail ASO, and following Snail-AAV treatment. Following Snail ASO treatment the levels and distribution of astrocytic connexins expression were reduced for both analyzed connexins. Snail-AAV showed an increase in Cn× 30 and a decrease in Cn× 43. c) Diagram of injured cortex showing the demarcation of the lesion core (0–150 μm) and peri-lesioned areas (150–500 μm) and scan line patterns through these areas. Plots for fluorescent intensity measurement for Connexin30, Connexin 43, and GFAP d) using line-scanning quantification distance-dependent from the center of the lesion (0–150 um) and peri-lesioned cortical areas (150–500 μm). Data are presented as a ratio, Kruskal Wallis + posthoc comparisons. *P* < 0.0001. Scale bars, low-magnification 5 μm, and high-magnification: 100 μm.

Previous studies suggested that activated microglia regulate connexins expression in astrocytes (61, 62), inhibiting astrocyte gap junctional communication (61) through pro-inflammatory cytokines (62), and modulating astrocyte phenotypes in disease (63). To determine whether Snail influenced gap junction protein expression in astrocytes in the current model, we analyzed changes in the main astrocyte connexins (C × 43 and C × 30) after injury and Snail ASO or AAV treatment. In the lesioned group, connexin 30 and 43 were upregulated at 7 dpl (Fig. 7a, b), with a uniform distribution on astrocytes processes. By contrast, following ASO Snail knockdown, an uneven distribution of connexins on astrocyte processes in the peri-lesioned areas was seen, while after AAV-induced Snail overexpression astrocytic connexins were selectively upregulated (Fig. 7a, b). Changes in connexin expression were spatially different relative to the lesion center. Line scan quantification by single-photon counting in two different areas related to injury track proximity (Fig. 7c) showed that at the center of the lesion (track and 150 mm adjacent) both C × 30 and C × 43 were significantly decreased after Snail-ASO (−20% and −36%, respectively) (Fig. 7d). Peri-lesioned areas (150–500 um) showed a less pronounced, decrease of astrocyte connexins following Snail ASO, suggesting that the effects of Snail were stronger closer to the center of the lesion. Overexpression of Snail by AAV resulted in an upregulation of C × 30 in the lesion and peri-lesioned areas (Fig. 7a and d), while C × 43 showed a decrease similar to that seen after ASO Snail knockdown. The differential distribution of connexin expression did not simply reflect astrocyte location/density changes. For example, analysis of GFAP expression showed increases relative to controls that were not lesion-distance dependent in Snail ASO samples (Fig. 7f). Together these results suggest that knockdown of Snail following CNS injury downregulates astrocyte connexins that may impair astrocyte network communications and modulate neuronal activity.

### Snail ASO treatment decreased neuronal survival and reduced postsynaptic density remodeling in the adult lesioned cortex

**Fig. 8.**
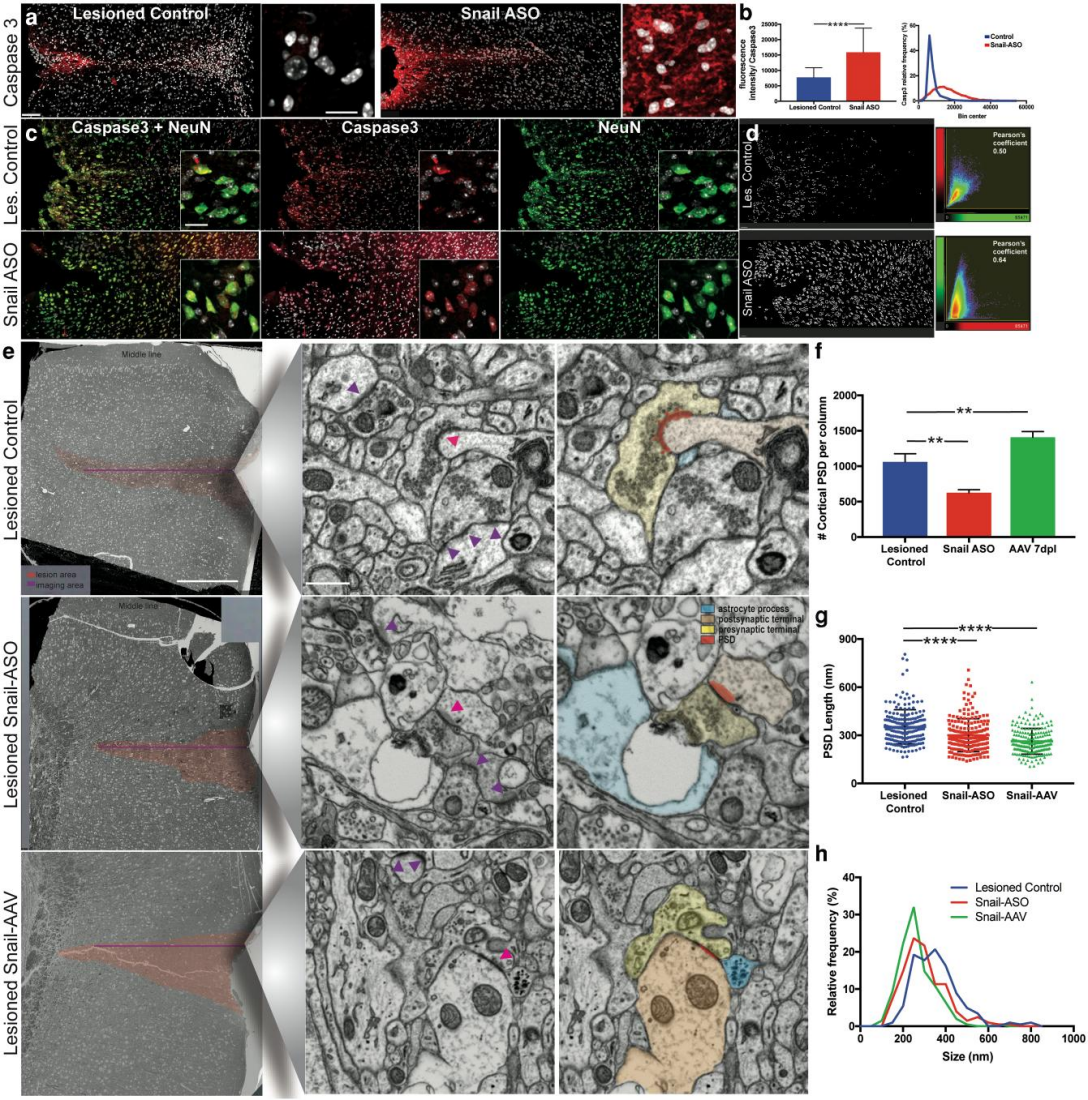
Snail expression postinjury affected neuronal survival and cortical synapses in the lesioned area. Snail ASO treatment affects neuronal survival after cortical injury. a) Representative confocal fluorescent tile images showing Caspase 3 expression in lesioned tissue and after Snail-ASO. b) Quantification of Casp-3 fluorescent intensity, data presented as mean ± SD, Mann-Whitney *P* < 0.0001, two-tailed. c) An increase in Casp-3 after ASO treatment is linked to NeuN expression. d) Colocalization channels and two-dimensional histograms for NeuN/Casp3 showed an increase after Snail-ASO. e) 2D-SEM overview of cortical lesion tracks (red silhouette) in control, after Snail ASO, and Snail-AAV treatment. The line in (e) represents ultrastructural cortical tile-imaged areas at high resolution (80.000×) along with all lesioned layers where synaptic quantification was performed. Inset of a single representative field of view showing active postsynaptic densities (arrowheads) in lesioned neuropil and false-color code of a tripartite synapse example for each group. f) Postsynaptic densities (PSD) quantification in lesioned, Snail-ASO, and Snail-AAV treated cortices. Data presented as mean ± SEM (Kruskal–Wallis + Dunn's posthoc comparisons). g) Graph bar for PSD length measurements in lesioned, Snail-ASO, and Snail-AAV treatment, data presented as mean ± min to max values (Kruskal–Wallis + Dunn's posthoc comparisons, *P* < 0.0001) and h) histograms showing changes in the PSD length distribution after Snail-ASO and Snail-AAV treatments. Scale bars: Low-mag→a–c: 50 μm and e–f: 500 nm. High-mag→a–c: 20 μm and e–f: 500 nm.

In other systems, the expression of Snail has been proposed to suppress caspase-mediated apoptosis (64, 65) and modulate the loss of selected neurons in C. elegans (66). In the current study, the lesioned controls had increased caspase-3 activation along the lesion track at 7 dpl, with the highest expression close to the pial surface (Fig. 8a). Following Snail-ASO knockdown caspase-3 expression was dramatically upregulated with fluorescent intensity analysis showing a 104% increase compared to the lesioned controls (Fig. 8a, b), suggesting loss of Snail results in increased cell death. Colocalization analysis indicated an increase in the degree of channel correlation for caspase-3 with the neuronal nuclei marker (NeuN) (Fig. 8c, d) but not between IBA1 and Casp3 (data not shown), suggesting an increase in neuronal apoptosis after Snail knockdown.

A specific population of astrocytes and microglia are intimately associated with the formation, maintenance, and removal of synapses (67, 68). Additionally, subsets of reactive astrocytes fail to support synapse formation or function (8) and activated microglia engage in synaptic pruning or stripping in pathological conditions. Postsynaptic densities also change their structure in response to synaptic activity (69). Since Snail modulation affected glial responses, we examined whether Snail affected local synapse organization, using high-resolution tiling-SEM images. The number of active postsynaptic densities in all cortical layers parallel to the injury track was compared between lesioned, ASO, and AAV-treated animals (Fig. 8e). Following Snail ASO knockdown alterations in the ultrastructural organization of perisynaptic astrocyte processes (Fig. 8e) were seen, including increases in the thickness in surrounding neuropil and retraction from perisynaptic sites compared to the lesioned group, consistent with tripartite synapse perturbation. By contrast, following Snail-AAV overexpression, thin fine distal processes of perisynaptic astrocytes filled with puncta-size debris were seen. Quantification of the density of postsynaptic densities adjacent to the lesion showed a significant decrease (41%) after Snail-ASO compared to lesioned controls (Fig. 8f). While AAV-induced Snail overexpression resulted in an increase of 53% in the density of cortical PSDs compared to lesioned controls (Fig. 8f). The relative size of the remaining PSDs was also affected. Following both Snail ASO knockdown and AAV-induced Snail overexpression a reduction of 14.72% in the length of PSDs was seen compared to lesioned controls (Fig. 8g, h). These data suggest that while the density of remaining PSDs after cortical injury may be influenced by levels of Snail, the regulation of the size of remaining PSDs is independent of Snail expression.

## Discussion

Multiple cell types contribute to neuroinflammatory responses following insults and injury to the CNS, including microglia and astrocytes. However, our understanding of the complex cellular interactions regulating glial responses to CNS insults is incomplete. Here, we identify a unique microglia-like cell population (IBA1^++^/CD86^++^/CD206^+^) induced in response to a cortical insult in the adult CNS. These cells are characterized by a dark cytoplasm, a high number of inclusions, and the expression of Snail, an EMT-transcriptional factor extensively studied for its role as a transcriptional repressor (70, 71). Our data suggest that Snail expression modulates CNS injury responses reducing glial reactivity and promoting neural protection. Following a cortical insult, overexpression of Snail results in reactive astrocyte phenotypes favoring a beneficial environment for neuron survival and connectivity. By contrast, inhibition of Snail expression results in decreased neuronal survival, elevated expression of pro-inflammatory cytokines, increased phagocytic astrocytes, reduced connexins expression, and perturbation of local synaptic structure. Our findings support the hypothesis that the expression of Snail in a subset of microglia/macrophage acts as a repressor of pro-inflammatory cytokines that enhance the induction of detrimental reactive astrocytes after injury, thereby promoting neuronal survival and reducing functional loss.

### Unique ultrastructure and molecular profile of Snail+ cells in the adult CNS

Based on the effects of modulating Snail expression, we propose that EMT-transcription factors and activators induced in the adult CNS in response to injury reflect a wound-healing response limiting secondary damage. This hypothesis is consistent with other studies in which EMT has been implicated in tissue fibrosis (72, 73), cutaneous (74, 75), prostatic (76), and ocular wound healing (77). In the CNS Zeb2, another EMT-transcription factor is expressed in astrocytes and regulates astrogliosis after CNS injury (78). In addition, neuroinflammation has been proposed to induce the expression of EMT triggers such as TGFb (79), WNT/β-catenin (80, 81), and Notch (82) in microglia/macrophages. Unlike in other tissues where the EMT response results in full or partial transdifferentiation of epithelial or epithelial-like to a mesenchymal phenotype, in the current CNS injury model, the expression of Snail in IBA1^+^/CD86^+^ cells promote a neuroprotective phenotype in glial cells without a complete phenotype transition, supporting the concept that EMT reflects a spectrum of the repair process and intermediary cell phenotypes (9).

In the absence of an insult, the adult CNS is largely devoid of Snail+ cells, and their localization close to the site of injury suggests they are injury-induced (48). Similarly, in vitro studies of the gastrointestinal tract showed that peripherally Snail is expressed in activated macrophages only at the site of injury or inflammation (46). The origin of the Snail+ cells in the injured CNS is unclear. They have a unique ultrastructure, with a darker cytoplasmic similar to that described in a subset of microglia under pathological conditions (47). Compared to dark microglia, however, the cytoplasm of activated Snail+ cells was larger in area and filled with a high density of large dense-core structures resembling lysosome inclusion bodies, and darker mitochondria consistent with enhanced phagocytotic properties. The expression of phenotypic markers has been controversial when used to distinguish functionally divergent microglia polarization (83), and while Snail+ cells had a high colocalization with IBA1 and the pro-inflammatory marker CD86 (84), they also showed a low colocalization with P2Y12, a marker for non-activated microglia (data not shown), suggesting a microglial origin. It is also possible, however, that infiltrating macrophages express Snail in response to injury. Indeed, it has been reported that peripheral macrophages in response to chronic neuroinflammation acquire specific microglia markers (85). Similarly, Snail may be expressed by infiltrated meningeal macrophages. After an injury, meningeal cells migrate into the lesion site after undergoing an epithelial–mesenchymal transition to repair the impaired meninges (48), and it has been shown that AKAP12 is expressed in arachnoid cells of the meninges, overlapping with fibronectin and ERTR7 (markers for meningeal cells). The same study showed an increase of Snail in the lesioned area opposite to AKAP12 expression 7 days after phototrombosis (48), making it unlikely that Snail cells come from a meningeal origin. Regardless of their origin, the injury-induced Snail+ CNS cells appear to play an important role in the current model by resolving inflammation and protecting the brain parenchyma from secondary damage. Furthermore, unlesioned control brain and AAV-Snail injected, showed a very limited Snail expression in the cortex (data not shown), suggesting that resting microglia/macrophage do not begin to express a significant level of Snail in the uninjured adult CNS, and/or that an additional injury-induced signal is required to fully express Snail.

The finding that Snail expression influences cytokine pathways that regulate downstream glial activation is consistent with data from other systems. For example, in monocytes derived from acute leukemia (THP-1), Snail expression is a key regulator of macrophage polarization induced by TGF-β (43), where overexpression in vitro promotes an anti-inflammatory phenotype while silencing of Snail results in a pro-inflammatory macrophage phenotype characterized by increased expression of CD86 and pro-inflammatory cytokines. Likewise, SNAIL1 has also been implicated in modulating the secretion of cytokines in an inflammatory breast cancer microenvironment (55). In the current study, the knockdown of Snail by ASO treatment increased cytokines known to stimulate the induction of neurotoxic astrocytes, while AAV-mediated snail overexpression, possibly acting as a transcriptional repressor of cytokine production, resulted in a decrease in cytokines that induce a neurotoxic astrocyte phenotype and a concomitant increase in S100A10-marker for neuroprotective astrocytes. Modulation of Snail expression affected multiple astrocyte characteristics. Knockdown of Snail by ASO treatment resulted in a downregulation of astrocytic connexins (C × 43 and 30) and Aqp4 expression suggesting a perturbation in astrocyte-astrocyte communication. Connexins and Aqp4 have been implicated in the phenotypic conversion of glial scar formation and neuroprotective astrocytes. C × 30 is required for optimal induction of neuroprotective astrocytes in Parkinson models (63), while C × 43 deletion increases astrogliosis and limits microgliosis after a stab wound (86). An interdependence between connexins and Aqp4 has been proposed (87), where deletion of astrocytic connexins leads to reduction and redistribution of perivascular Aqp4 consistent with the results of the current study. Based on these data it seems likely that changes in the phenotype of reactive astrocytes after Snail knockdown affect the regulation of astrocytic Cxs/Aqp4 resulting in an enhanced glial response.

The changes in astrocyte characteristics resulting from the knockdown of Snail expression likely contribute to increased cellular loss and secondary damage after CNS injury. The increase in neuronal cell death seen after Snail knockdown suggests that it is neuroprotective after adult CNS insults, possibly through regulating reactive astrocyte phenotypes. Recent studies suggest that activated microglia and reactive astrocytes disrupt cortical synapses compromising information flow in the CNS (8), and the reduction in the number of synapses in regions adjacent to the lesion ASO is consistent with elevated glial responses. One potential mechanism to explain the synaptic reduction is that reductions in Snail expression increase activation of complement-mediated synaptic pruning/engulfment based on enhanced C1q upregulation postinjury. C1q is the initiating protein in the classical complement cascade, expressed by microglia in the injured adult brain, and localized at synapses tagged for elimination (88). Consistent with this hypothesis, the expression of C1q was increased following Snail knockdown. Conversely, Snail overexpression decreased C1q expression resulting in less selectively tagged synapses for elimination in the lesioned area. The increased neuronal loss following Snail ASO treatment may also result in fewer neurons and axons/spines to ensure appropriate synaptic communication, and in either case, compromising the recovery of the cortical neural circuitry after injury. Our data also suggested that Snail is playing a pivotal role in mediating astrocytic phagocytosis postinjury, where the loss of Snail in microglial is associated with an increase in phagocytic astrocytes engulfing large cell debris, instead of the smaller size of cell debris characteristic of noninjury associated phagocytic astrocytes. Similarly, after Snail overexpression, the phagocytic inclusions are very sparse, comparable to a reactive state astrocyte. While the astrocytic glycogen accumulation is higher, even in the fine distal processes intermingled with the neuropil and synapses, consistent with an increase in glycogen mobilization that could exert a neuroprotective effect against CNS damage (56). Taken together our data indicate that following a cortical insult, there is an induction of a unique population of Snail+ cells in the adult CNS, and snail modulation results in changes in glial responses suggesting that Snail expression serves to mitigate further damage following insults to the adult CNS.

## Materials and methods

### Experimental animals and surgical procedures

All animal procedures were approved and conducted in accordance with the guidelines of The George Washington University Office of Animal Research. Animals (10–12 weeks old) female C57BL/6J wild-type mice (Charles River) were distributed into nonlesioned, lesioned+ control-ASO, lesioned + Snail-ASO, lesioned + control-AAV, and lesioned + Snail-AAV. Responses were assessed at 7 days postlesion.

Following anesthesia [isoflurane 2%], animals underwent a unilateral cortical stab wound. A stereotaxically guided needle was inserted (z = 600–800 μm) into the right hemisphere (dorsoventral/mediolateral −1.2 mm from Bregma), with a rostrocaudal extension of 400 μm. The lesioned areas were mainly restricted to gray matter, affecting neocortical columns with less white matter (corpus callosum) damage. The lesioned ASO groups received an intracortical injection of control or Snail ASO [2μL, 53.45 ng/μL] (11 Elite Nanomite, Harvard apparatus), with a flow rate of 50 nl/sec, followed by a 2-day regime of cisterna magna injection with ASO [2 μL] in the cerebrospinal fluid. For experiments in which the effect of Snail overexpression after injury was assessed, we delivered in vivo 50 nl SNAI1 AAV at a titer of 2.3 × 10^13^ GC/mL via cisterna magna 21 days before the cortical injury, followed by a second injection 7 days before the cortical lesion approach. All animals received institutional animal care and use committee-approved prophylactic and postsurgical pain management. No long-lasting side effects were detected in any animals' health or survival rate.

### Snail antisense oligonucleotides (Snail-ASOs)

To silence the expression of Snail, four different in vivo locked nucleic acids (LNA) GapmeR antisense oligonucleotides were designed and screened (See Supplementary Table S1) using Exiqon’s locked nucleic acid GapmeR algorithm design tool (Qiagen). All Snail-ASOs were single-stranded (16 nucleotides long) with a different target in the *Snai1* mouse sequence and enriched with LNA-containing flanking regions to improve the binding affinity and thermal stability. ASOs contained backbone modifications (phosphorothioate) to improve resistance to enzymatic degradation. After the screening, one Snail-ASO was tagged with fluorescein to analyze its distribution following CNS injection. A predesigned ASO-negative control RNA (Qiagen, cat #1027295) with an Alexa Fluor 647 modification was used to control for nonspecific ASO effects. No changes in the expression of Snail were detected following the use of the control ASO.

### Mouse SNAI1 over-expression adeno-associated virus (AAV)

To enhance in vivo Snail expression in the lesioned cortex of the mice, a commercial recombinant adeno-associated virus designed to over-express mouse SNAI1 (Vector Biolabs AAV-272698) was customized using a microglia-specific F4/80 promoter, a PHP.eB capsid specific for CNS applications, allowing blood-brain barrier crossing, and tagged to mCherry reporter to validate transcription specificity. The SNAI1 AAV was used in combination with a control AAV provided by the same company. The titer of the viral stock for the SNAI1 vector was 3.4 × 10^12^ (genomic copies per mL) GC/mL measured by qPCR (bGH polyA). We successfully conducted an in vitro pilot testing for the customized SNAI1 AAV specificity infecting microglia/macrophages in mixed cell cultures for 3 days and overexpressing Snail (data not shown). No changes in the expression of Snail were detected with control AAV. For in vivo studies, animals survived 7 days after cortical injury, followed by transcardial perfusions 28 days after the first SNAI1 AAV was delivered.

### Immunohistochemistry

For immunohistochemistry, animals were perfused transcardially with 4% paraformaldehyde (PFA) in 0.1 M phosphate buffer (PBS) and brains were postfixed overnight at 4°C, cryopreserved, embedded in optimal cutting temperature compound (OCT), and the cortical region containing the lesion sectioned coronally (14 μm). For each analysis, simultaneously standard immunohistochemical protocols for all groups were performed. In addition, we matched the same level of the lesions for all groups. Briefly, sections were washed with tris buffered saline–Triton (TBST) X100 0.03% (0.05 M, pH 7.6) and blocked using 10% normal goat serum (1 hour, room temperature/RT). Sections were incubated with specific primary antibodies/concentrations (Supplementary Table S2) overnight at 4°C and then washed and incubated (1 hour at RT) in the corresponding Alexa Fluor secondary antibodies [1:500] in TBST. To perform Snail labeling, we used a modified heat-induced epitope retrieval method (89). To test the specificity of the detection system, incubation with primary antibodies was omitted and no specific staining was observed. Positive controls for Snail expression included analysis in mouse embryonic tissue (data not shown). All immunofluorescence data were collected using a multiphoton confocal microscope (TCS-SP8; Leica Microsystems) or Zeiss spinning disk confocal with high numerical aperture and multi-immersion corrected objective lenses (40×/1.04, 100×/1.46). To deliver a robust and accurate quantification, we used hybrid detectors (HyDs) in photon counting mode (binary events: photon or no photon), to provide a straightforward path for intensity fluorescent measurements. All images to perform a comparison between groups (fluorescent intensity) were taken using the same optimized imaging parameters for exposure time, gain, laser illumination power, and z spacing.

### Electron microscopy preparation and scanning electron microscopy (SEM)

To analyze ultrastructural changes, animals from both groups were perfused transcardially with 4% PFA and 1% glutaraldehyde (GA) in 0.1 M phosphate buffer (PB). Coronal brain slices (200 μm) were vibratome-sectioned and incubated in 3% potassium ferrocyanide in 0.3 M cacodylate buffer with 4 mM calcium chloride and 4% aqueous osmium tetroxide (1 hour, 4°C) followed by a filtered thiocarbohydrazide solution (20 min/RT), washed, and incubated in an aqueous 2% osmium tetroxide solution (30 min/RT). Sections were placed overnight in 1% uranyl acetate (4°C) and stained *en bloc* using Walton's lead aspartate solution. Samples were dehydrated through graded ethanol series, infiltrated with epoxy resin (EMbed-812; Electron Microscopy Science, Hatfield, PA, USA), and embedded between thermoplastic fluoropolymer films (Aclar, EMS). Blocks containing cortical lesions were sectioned using an ultramicrotome (z = 120 nm; UC7 Leica Microsystems), ultrathin sections were placed in silicon wafers and carbon-taped in aluminum stubs for SEM imaging in a Helios NanoLab 660 FIBSEM (ThermoFisher). To maximize the collection of the backscattered electrons, we used a concentric detector in immersion mode at a 4-um working distance, using 2 kV and 0.40 nA current landing. Overviews of the entire cortical areas were performed in low magnification (1000×) to identify the lesioned areas, and then high-resolution tile images of desired structures were performed using 80.000 × magnification (dwell time:5μs, 3072 × 2,048 resolution) with a pixel size of 1.6862 (MAPS 3.16, ThermoFisher).

### Correlative light microscopy and focus ion beam scanning electron microscopy (FibSEM)

To assess the ultrastructural characteristics of Snail+ cells, fluorescent data were merged with 3D-SEM ultrastructural milling 3D reconstruction. Following regular immunohistochemistry for DAPI/Snail/GFAP primary antibodies doubling incubations time, sections were analyzed by fluorescence confocal imaging using a multiphoton microscope TCS sp8 (Leica Microsystems), combining pulsed white laser and hybrid detectors, the lesioned zones were imaged with DFC365FX camera at a resolution of 2048 × 2,048 pixels using a 20 × (NA 0.75) oil immersion object. After imaging, samples were incubated overnight (4°C) in PFA (4%) and GA (1%) and were prepared for SEM as described above.

Imaged cortical sections were trimmed and block-mounted in pin stubs. The sample was approached with the ultramicrotome to remove excess plastic and then covered with conductive silver paint (Ted Pella) to maximize conductivity. To decrease sample charging, the sample was sputter-coated with an iridium layer (1 nm) and then large-field imaged using a Helios NanoLab 660 SEM equipped with a concentric backscatter detector in immersion mode (2 kV 0.40 nA, 5 μs dwell time, horizontal field of view/HFV; 20.3 um), obtaining an entire high-resolution tile stitching overview of the cortical lesion and adjacent areas. This overview was used to correlate positive Snail cells from fluorescent confocal stacked images with reference to fiducial structures (blood vessels, astrocytes processes, and lesion track) in the SEM tile-stitching overview. After identification of the Snail+ cell in the SEM tile-stitched overview, Gallium beam-induced platinum layer deposition was performed as a protective layer. Surplus block material was removed with a high ion beam current (30 kV, 6.5 nA) (see Fig. 2c). For final surface polishing/milling, we used a reduced ion current (30 kV, 2.8 nA). For imaging, the Auto Slice and View G4 software (FEI, Thermo Fisher) was used for fully automated acquisition with an electron beam current of 400 pA, kV 2.0, and horizontal field width of 11.84 μm to acquire an image stack of 154 sections (pixel size, 1.97; z = 50 nm) using a through-the-lens detector, with a resolution of 6,144 × 4,096, dwell time of 6 μs, and a working distance of 4.04 mm. Volumetric fluorescent reconstructions and SEM Snail cell 3D reconstructions were performed using Imaris (9.5.1).

### Protein fluorescence colocalization/quantification and angle measurement of GFAP positive profiles

To visualize the correlative light and SEM data and to segment/quantify protein expression changes, raw files from confocal images were migrated in Imaris (9.5, Bitplane). For correlative light and electron microscope (CLEM) analysis, a surface model was used to reconstruct the volumes of two types of imaging modalities. New surfaces per channel were created for fluorescence intensity quantification to segment and quantify the labeling (Imaris 9.5). We performed background subtraction to define the background at each voxel, and then a baseline subtraction of this variable was done using Gaussian filtering. Thresholds were selected by visual inspections while filtering (number of voxels) was applied to create each surface model. Surface editing was performed to delete the remaining background labeling, and then a mask channel was created to extract the intensity information for their corresponding Alexa Fluor channel. In each new mask channel, the statistic values per surface (intensity mean) and the intensity histograms were extracted automatically.

To analyze changes in the presence of two fluorochromes at the same location, we used the 3D colocalization analysis tool (ImarisColoc) following the method described by Costes et al. (90), with masked channel selection and automatic calculation of Pearson's and Mander's coefficient. A colocalization channel was built to illustrate the colocalization of the fluorescent information and 2D-histograms, reflecting the distribution of pairs of voxel intensities occurring in the two selected channels. To evaluate alterations in the astrocyte features after Snail-ASO and Snail-AAV, an automated line scan quantification from two-photon images in labeled sections for connexins and GFAP was utilized using HyD detectors (super-sensitive imaging), maximizing the dynamic resolution by quantification through single-photon counting on two different areas per sample: (i) 0–150 μm from the center of the lesion- core zone (Fig. 7c) and (ii) 151–500 μm (peri-lesioned zone), 50 bilateral lines were traced and quantified per sample and protein in 2D maximum projection from 3D-confocal images, all the data was obtained using intuitive software control by LASX software (Leica Microsystems), the numerical labeling results were collected and analyzed using Prism 7.

## Supplementary Material

pgad334_Supplementary_Data

## Data Availability

All research data is available in the main text or supplementary material.
