# Supplementary material for: A unique cell population expressing the Epithelial-Mesenchymal Transition-transcription factor Snail moderates microglial and astrocyte injury responses
Source: PNAS Nexus. 2023 Oct 12;2(10):pgad334. doi: 10.1093/pnasnexus/pgad334 (PMC10612478; doi:10.1093/pnasnexus/pgad334)
Supplement: pgad334_Supplementary_Data [file pgad334_supplementary_data.zip › PNASNEXUS-PNASNEXUS-2023-00964-T-s04.docx]

**Table S2. Primary Antibodies**

| **1ry AB** | **Host species** | **Catalog #** | **Concentration** |
| --- | --- | --- | --- |
| Snail | Rabbit | Invitrogen/MA5-14801 | 1:200 AR* |
| Slug | Rabbit | Abcam/ ab27568 | 1:500 no AR |
| ZEB1 | rabbit | Sigma/SAB2102759 | 1:300 no AR |
| TWIST2 | Mouse | Proteintech/66544-1 | 1:50 no AR |
| B-catenin | Rabbit | Abcam/ab224803 | 1:100 no AR |
| Vimentin | Rabbit | Abcam/ab92547 | 1:250 no AR |
| IBA1 | Rabbit | Wako/ 019-19741 | 1:500 no AR |
| GFAP | Rabbit | DAKO/Z0334 | 1:500 no AR |
| GFAP | Rat | Invitrogen/ 13-0300 | 1:500 no AR |
| NeuN | Mouse | Millipore Sigma/ MAB377 | 1:250 no AR |
| Anti-CD13 | Rabbit | abcam/EPR4058 | 1:500 no AR |
| CD31/PECAM | Rat | Novus/ NB600-1475 | 1:500 no AR |
| CD86 (B7-2) | Rat | ThermoFisher/14-0862-82 | 1:500 AR |
| MMR/CD206 | Goat | R&D systems | 1:100 no AR |
| Anti-C1q | Rabbit | abcam/ ab182451 | 1:500 AR |
| Anti-TNF alpha | Mouse | abcam/ ab1793 | 1:500 AR |
| IL-1 alpha | Rabbit | Abcam/ ab9724 | 1:500 AR |
| P2RY12 | Rat | BioLegend/848001 | 1:500 AR |
| Connexin 30 | Rabbit | ThermoFisher/ 71-2200 | 1:500 no AR |
| Connexin 43 | Rabbit | Millipore Sigma/ C6219 | 1:500 no AR |
| PDGF R beta | Mouse | R&D systems/ AF1042 | 1:500 no AR |
| AQP4 | Rabbit | Millipore Sigma/A5971 | 1:100 no AR |
| Caspase3 | Mouse | BioLegend/ 622701 | 1:500 no AR |
| iNOS | Rat | BioLegend/696802 | 1:500 AR |

*AR: Antigen retrieval + Tween
