# Supplementary material for: A unique cell population expressing the Epithelial-Mesenchymal Transition-transcription factor Snail moderates microglial and astrocyte injury responses
Source: PNAS Nexus. 2023 Oct 12;2(10):pgad334. doi: 10.1093/pnasnexus/pgad334 (PMC10612478; doi:10.1093/pnasnexus/pgad334)
Supplement: pgad334_Supplementary_Data [file pgad334_supplementary_data.zip › PNASNEXUS-PNASNEXUS-2023-00964-T-s05.docx]

**Table S1. Antisense Oligonucleotides**

| **Product Name** | **Sequence direction** | **Sequence** | **Mw. Calc. (Da)** |
| --- | --- | --- | --- |
| SP/Q02085/SNAI1_MOUSE_5 (*) | 5'-3' (fluorescein tag) | AAGATGCCAGCGAGGA | 5345.26 |
| SP/Q02085/SNAI1_MOUSE_3 | 5'-3' | GGGAAACGGGTAATCT | 5323.24 |
| SP/Q02085/SNAI1_MOUSE_2 | 5'-3' | GTGGACGAGAAGGACG | 5429.33 |
| SP/Q02085/SNAI1_MOUSE_1 | 5'-3' | ATTACCAGCCTTGAAT | 5221.18 |

(*) after the screening, this was the most effective Snail ASO and was used for all the subsequent experiments.
