# Supplementary figures and images for: A unique cell population expressing the Epithelial-Mesenchymal Transition-transcription factor Snail moderates microglial and astrocyte injury responses
Source: PNAS Nexus. 2023 Oct 12;2(10):pgad334. doi: 10.1093/pnasnexus/pgad334 (PMC10612478; doi:10.1093/pnasnexus/pgad334)

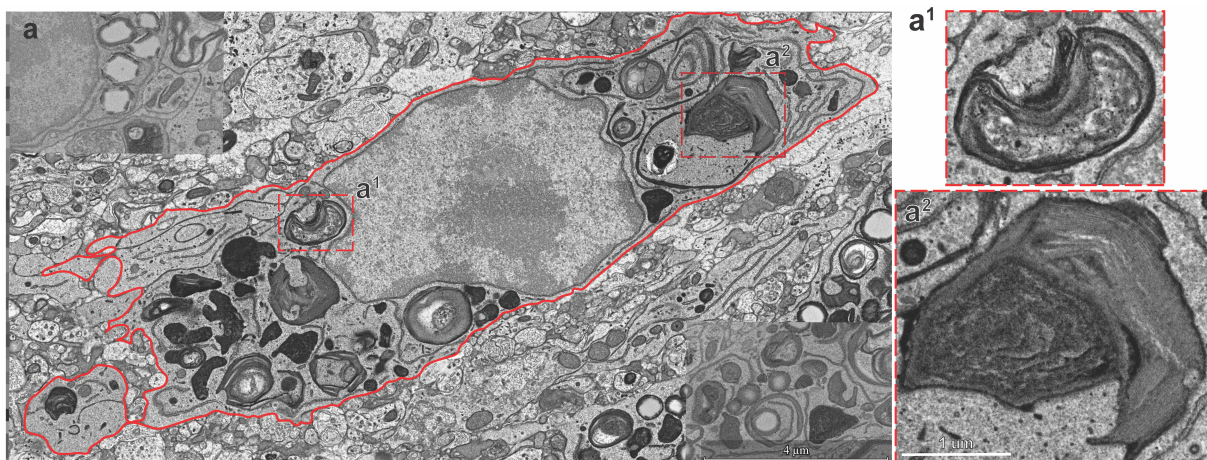

Supplement: pgad334_Supplementary_Data [file pgad334_supplementary_data.zip › PNASNEXUS-PNASNEXUS-2023-00964-T-s01.pdf]
